# C-reactive protein to albumin ratio predicts survival in patients with unresectable hepatocellular carcinoma treated with lenvatinib

**DOI:** 10.1038/s41598-022-12058-y

**Published:** 2022-05-19

**Authors:** Toshifumi Tada, Takashi Kumada, Atsushi Hiraoka, Masashi Hirooka, Kazuya Kariyama, Joji Tani, Masanori Atsukawa, Koichi Takaguchi, Ei Itobayashi, Shinya Fukunishi, Kunihiko Tsuji, Toru Ishikawa, Kazuto Tajiri, Hironori Ochi, Satoshi Yasuda, Hidenori Toyoda, Takeshi Hatanaka, Satoru Kakizaki, Noritomo Shimada, Kazuhito Kawata, Takaaki Tanaka, Hideko Ohama, Kazuhiro Nouso, Asahiro Morishita, Akemi Tsutsui, Takuya Nagano, Norio Itokawa, Tomomi Okubo, Taeang Arai, Michitaka Imai, Atsushi Naganuma, Tomoko Aoki, Yohei Koizumi, Shinichiro Nakamura, Kouji Joko, Yoichi Hiasa, Masatoshi Kudo

**Affiliations:** 1 Department of Internal Medicine, Japanese Red Cross Himeji Hospital, 1-12-1 Shimoteno, Himeji, Hyogo 670-8540 Japan; 2 Department of Nursing, Gifu Kyoritsu University, Ogaki, Japan; 3 Gastroenterology Center, Ehime Prefectural Central Hospital, Matsuyama, Japan; 4 Department of Gastroenterology and Metabology, Ehime University Graduate School of Medicine, Touon, Ehime Japan; 5 Department of Gastroenterology, Okayama City Hospital, Okayama, Japan; 6 Department of Gastroenterology and Hepatology, Kagawa University, Kagawa, Japan; 7 Division of Gastroenterology and Hepatology, Department of Internal Medicine, Nippon Medical School, Tokyo, Japan; 8 Department of Hepatology, Kagawa Prefectural Central Hospital, Takamatsu, Japan; 9 Department of Gastroenterology, Asahi General Hospital, Asahi, Japan; 10 Department of Gastroenterology, Osaka Medical College, Osaka, Japan; 11 Center of Gastroenterology, Teine Keijinkai Hospital, Sapporo, Japan; 12 Department of Gastroenterology, Saiseikai Niigata Hospital, Niigata, Japan; 13 Department of Gastroenterology, Toyama University Hospital, Toyama, Japan; 14 Hepato-Biliary Center, Japanese Red Cross Matsuyama Hospital, Matsuyama, Japan; 15 Department of Gastroenterology and Hepatology, Ogaki Municipal Hospital, Ogaki, Japan; 16 Department of Gastroenterology, Gunma Saiseikai Maebashi Hospital, Maebashi, Japan; 17 Department of Clinical Research, National Hospital Organization Takasaki General Medical Center, Takasaki, Japan; 18 Division of Gastroenterology and Hepatology, Otakanomori Hospital, Kashiwa, Japan; 19 Department of Hepatology, Hamamatsu University School of Medicine, Hamamatsu, Japan; 20 Department of Gastroenterology, National Hospital Organization Takasaki General Medical Center, Takasaki, Japan; 21 Department of Gastroenterology and Hepatology, Kindai University, Osaka, Japan

**Keywords:** Gastrointestinal cancer, Liver cancer

## Abstract

We investigated the impact of C-reactive protein to albumin ratio (CAR) on predicting outcomes in 522 patients with unresectable hepatocellular carcinoma (HCC) treated with lenvatinib. We determined the optimal CAR cutoff value with time-dependent receiver operating characteristic curve analysis. Additionally, we clarified the relationship between CAR and liver function or HCC progression. Median overall survival was 20.0 (95% confidence interval (CI), 17.2–22.6) months. The optimal CAR cutoff value was determined to be 0.108. Multivariate analysis showed that high CAR (≥ 0.108) (hazard ratio (HR), 1.915; 95% CI, 1.495–2.452), Eastern Cooperative Oncology Group performance status ≥ 1 (HR, 1.429), and α-fetoprotein ≥ 400 ng/mL (HR, 1.604) were independently associated with overall survival. Cumulative overall survival differed significantly between patients with low versus high CAR (*p* < 0.001). Median progression-free survival was 7.5 (95% CI, 6.7–8.1) months. Multivariate analysis showed that age, CAR ≥ 0.108 (HR, 1.644; 95% CI, 1.324–2.043), and non-hepatitis B, non-hepatitis C etiology (HR, 0.726) were independently associated with progression-free survival. Cumulative progression-free survival differed significantly between patients with low versus high CAR (*p* < 0.001). CAR values were significantly higher as Japan Integrated Staging score increased (*p* < 0.001). In conclusion, CAR can predict outcomes in patients with unresectable HCC treated with lenvatinib.

## Introduction

Hepatocellular carcinoma (HCC) is the most commonly encountered primary liver malignancy and the sixth most common malignant disease in the world^1^. Surgical resection, liver transplantation, and local ablation therapy are indicated for early-stage HCC^2^. For patients with intermediate and advanced HCC, transarterial chemoembolization and systemic therapies such as targeted therapy or immunotherapy are usually recommended^2^.

Sorafenib, a molecularly targeted agent, has been developed for first-line systemic treatment of patients with unresectable HCC^3,4^. In 2018, lenvatinib^5^, a newly developed tyrosine kinase inhibitor, became available in Japan as first-line therapy in patients with unresectable HCC. More recently in Japan, the combination of atezolizumab plus bevacizumab has been approved as first-line systemic therapy in patients with unresectable HCC^6^.

Various clinical markers such as age, sex, Eastern Cooperative Oncology Group performance status (ECOG-PS), α-fetoprotein, hepatic function, hepatic fibrosis, and HCC stage have been reported as predictors of survival in patients who underwent treatment for HCC^1,7–10^. However, characteristics associated with survival in patients with unresectable HCC treated with lenvatinib have not been sufficiently investigated.

High C-reactive protein (CRP) to albumin ratio (CAR) has been reported to be associated with poor outcomes in numerous malignancies^11–16^. This predictor of systemic inflammation is easy and inexpensive to measure^11–16^. Several reports have suggested that elevated pretreatment CAR might be associated with poor outcomes in patients with HCC treated with resection, radiofrequency ablation, or transarterial chemoembolization^15,16^. However, the association between CAR and prognosis in patients with unresectable HCC treated with molecularly targeted agents, especially lenvatinib, has not been studied.

In this study, we investigated the impact of CAR on predicting overall survival and progression-free survival in patients with unresectable HCC treated with lenvatinib using clinical data from multiple Japanese centers specializing in liver disease.

## Results

### Patient characteristics

The baseline characteristics of the 522 study patients are summarized in Table 1. There were 112 (21.5%) females and 410 (78.5%) males with a median age of 73.0 (68.0–79.0) years. Median CAR was 0.079 (0.027–0.254). There were 5 (1.0%) patients with Japan Integrated Staging (JIS) score 0, 88 (16.9%) patients with JIS score 1, 197 (37.7%) patients with JIS score 2, 226 (43.3%) patients with JIS score 3, and 6 (1.1%) patients with JIS score 4. Median follow-up was 13.3 (7.0–22.6) months. Table 1 also lists the baseline characteristics of the 522 study patients stratified by CAR level.

**Table 1:** Characteristics of the study patients.

	Overall	Stratified by CAR		
	(n = 522)	CAR < 0.108 (n = 302)	CAR ≥ 0.108 (n = 220)	*p* value
Age* (years)	73.0 (68.0–79.0)	74.0 (69.0–80.0)	72.0 (66.0–78.0)	0.039
Sex (female/male)	112 (21.5%)/410 (78.5%)	73 (24.2%)/229 (75.8%)	39 (17.7%)/181 (62.3%)	0.084
ECOG-PS (0/1/2/3)	397 (76.1%)/108 (20.7%)/16 (3.1%)/1 (0.2%)	234 (77.5%)/59 (19.5%)/8 (2.6%)/1 (0.3%)	163 (74.1%)/49 (22.3%)/8 (3.6%)/0 (0.0%)	0.668
Body mass index (kg/m^2^)	23.1 (20.9–25.5)	23.7 (21.3–25.4)	22.6 (20.6–25.5)	0.120
Etiology of HCC (hepatitis B/C/non-B, non-C)	74 (14.2%)/217 (41.6%)/231 (44.3%)	44 (14.6%)/142 (47.0%)/116 (38.4%)	30 (13.6%)/75 (34.1%)/115 (52.3%)	0.005
Albumin (g/dL)*	3.7 (3.3–4.0)	3.9 (3.6–4.1)	3.5 (3.0–3.8)	< 0.001
Total bilirubin (mg/dL)*	0.7 (0.5–1.0)	0.7 (0.6–1.0)	0.8 (0.5–1.1)	0.692
CRP (mg/dL) *	0.28 (0.11–0.89)	0.12 (0.06–0.22)	1.10 (0.66–2.39)	< 0.001
CAR*	0.079 (0.027–0.254)	0.032 (0.015–0.058)	0.322 (0.183–0.701)	< 0.001
α-fetoprotein (ng/mL)*	30.0 (5.6–691.1)	24.1 (5.3–390.3)	44.4 (6.3–1322.8)	0.090
Child–Pugh score (5/6/ ≥ 7)	302 (57.9%)/206 (39.5%)/14 (2.6%)	211 (69.9%)/87 (28.8%)/4 (1.3%)	91 (41.4%)/119 (54.1%)/10 (4.5%)	< 0.001
Macroscopic vascular invasion (yes/no)	115 (22.0%)/407 (78.0%)	55 (18.2%)/247 (81.8%)	60 (27.3%)/160 (72.7%)	0.018
Extrahepatic spread (yes/no)	192 (36.8%)/330 (63.2%)	99 (32.8%)/203 (67.2%)	93 (42.3%)/127 (57.7%)	0.028
BCLC stage (0/A/B/C/D)	4 (0.8%)/12 (2.3%)/218 (41.8%)/287 (55.0%)/1 (0.2%)	4 (1.3%)/9 (3.0%)/134 (44.4%)/154 (51.0%)/1 (0.3%)	0 (0.0%)/3 (1.4%)/84 (38.2%)/133 (60.5%)/0 (0.0%)	0.064
TNM LCSGJ 6th edition (I/II/III/IV)	5 (1.0%)/89 (17.0%)/203 (38.9%)/225 (43.1%)	5 (1.7%)/60 (19.9%)/125 (41.4%)/112 (37.1%)	0 (0.0%)/29 (13.2%)/78 (35.5%)/113 (51.4%)	0.002
JIS score (0/1/2/3/4)	5 (1.0%)/88 (16.9%)/197 (37.7%)/226 (43.3%)/6 (1.1%)	5 (0.1%)/60 (19.9%)/123 (40.7%)/112 (37.1%)/2 (0.7%)	0 (0.0%)/28 (12.7%)/74 (33.6%)/114 (51.8%)/4 (1.8%)	0.001
History of sorafenib therapy (yes/no)	124 (23.8%)/398 (76.2%)	55 (18.2%)/247 (81.8%)	69 (31.4%)/151 (68.6%)	0.001
Follow-up duration* (months)	13.3 (7.0–22.6)	16.9 (9.5–25.2)	9.6 (5.2–17.5)	< 0.001
Therapeutic response				0.134
Complete response	24 (5.0%)	16 (5.7%)	8 (4.0%)	
Partial response	178 (37.0%)	113 (40.4%)	65 (32.3%)	
Stable disease	189 (39.3%)	106 (37.9%)	83 (41.3%)	
Progressive disease	90 (18.7%)	45 (16.1%)	45 (22.4%)	
Not evaluated	41	22	19	
Overall response rate	42.0%	46.1%	36.3%	0.039
Disease control rate	81.3%	83.9%	77.6%	0.097

*ECOG-PS* Eastern Cooperative Oncology Group performance status; *HCC* Hepatocellular carcinoma; *CRP* C-reactive protein; *CAR* C-reactive protein to albumin ratio; *BCLC* Barcelona Clinic Liver Cancer; *TNM LCSGJ 6th* Tumor node metastasis stage according to the 6th edition of the Liver Cancer Study Group of Japan guidelines; *JIS* Japan Integrated Staging.

*Data expressed as medians (interquartile range).

### Overall survival and progression-free survival

**Figure 1:**
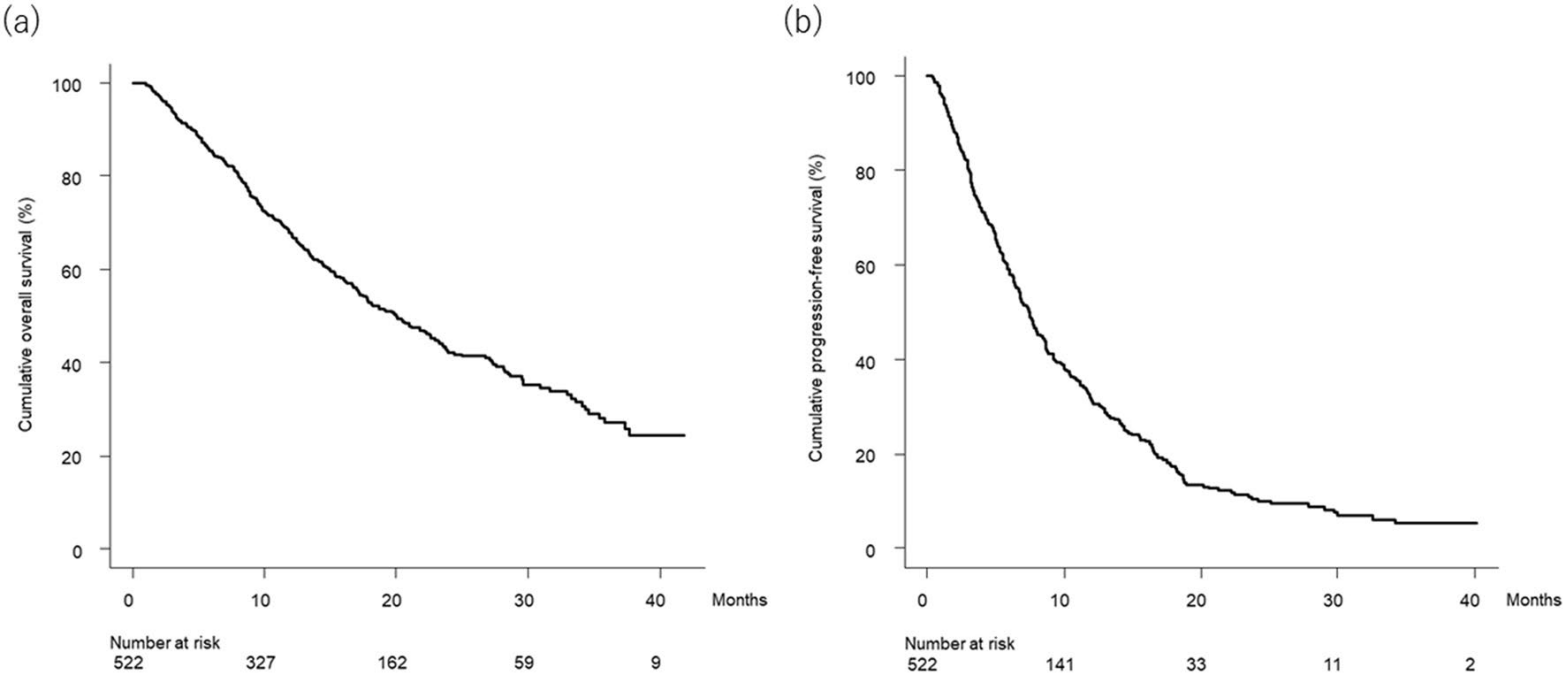
(**a**) Cumulative overall survival curve. Cumulative overall survival at 6, 12, 18, 24, 30, and 36 months was 85.4%, 67.6%, 52.7%, 42.2%, 35.2%, and 27.1%, respectively. (**b**) Cumulative progression-free survival curve. Cumulative progression-free survival at 3, 6, 9, 12, 15, and 18 months was 80.2%, 59.1%, 41.3%, 31.4%, 24.1%, and 17.4%, respectively.

Figure 1a shows the curve for overall survival in the study patients. Median overall survival was 20.0 (95% confidence interval [CI], 17.2–22.6) months. Figure 1b shows the curve for progression-free survival in the study patients. Median progression-free survival was 7.5 (95% CI, 6.7–8.1) months.

**Figure 2:**
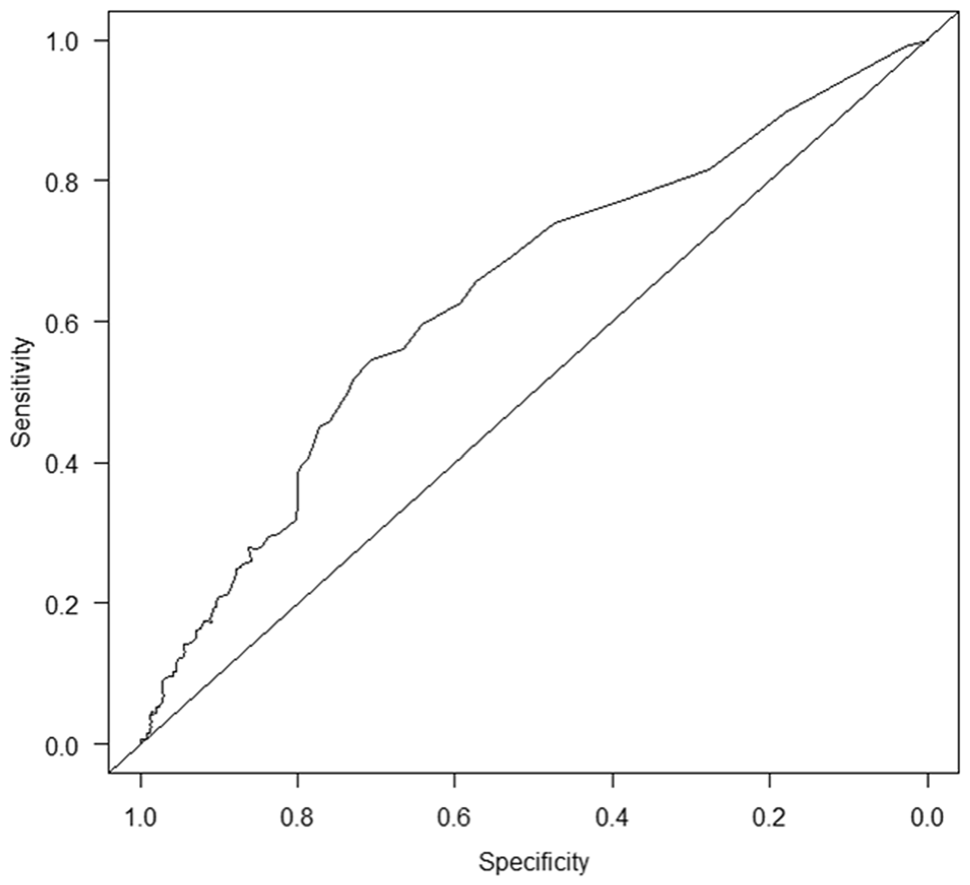
Time-dependent ROC curve of CAR for overall survival at 20 months after the start of follow-up. The area under the ROC curve was 0.638. Sensitivity and specificity using the CAR cutoff value of 0.108 according to the Youden index were 54.7% and 71.0%, respectively. ROC, receiver operating characteristic; CAR, C-reactive protein to albumin ratio.

Figure 2 shows the receiver operating characteristic (ROC) curve for CAR with overall survival at 20 months after the start of follow-up based on time-dependent ROC analysis. The optimal CAR cutoff value was determined to be 0.108 based on the Youden index.

### Therapeutic response

Radiological best response rates for complete response, partial response, stable disease, and progressive disease were 5.0% (24/481), 37.0% (178/481), 39.3% (189/481), and 18.7% (90/481), respectively. Therapeutic response was not evaluated in 41 patients. The overall response rate was 42.0% and the disease control rate was 81.3% (Table 1). Therapeutic responses stratified by CAR are also listed in Table 1. ORR was significantly different between patients with low CAR and high CAR (*p* = 0.039).

### Factors associated with overall survival

Factors associated with overall survival in the univariate analysis are listed in Table 2. The following variables were statistically significant: ECOG-PS, α-fetoprotein, extrahepatic spread, history of sorafenib therapy, and CAR.

**Table 2:** Overall survival analysis.

	Univariate analysis			Multivariate analysis		
	HR	95% CI	*p* value	HR	95% CI	*p* value
*Age (years)*						
< 75 (n = 303)	1	0.827–1.344	0.668	1	0.847–1.408	0.498
≥ 75 (n = 219)	1.055			1.092		
*Sex*						
Female (n = 112)	1	0.652–1.163	0.349	1	0.709–1.292	0.775
Male (n = 410)	0.871			0.957		
*ECOG-PS*						
0 (n = 397)	1	1.058–1.832	0.018	1	1.075–1.899	0.014
≥ 1 (n = 125)	1.392			1.429		
*Etiology*						
Viral hepatitis (n = 291)	1	0.657–1.071	0.159	1	0.614–1.015	0.065
Non-B, non-C (n = 231)	0.839			0.789		
*α-fetoprotein (ng/mL)*						
< 400 (n = 373)	1	1.384–2.285	< 0.001	1	1.237–2.079	< 0.001
≥ 400 (n = 149)	1.778			1.604		
*Macroscopic vascular invasion*						
Absent (n = 407)	1	0.920–1.612	0.168	1	0.734–1.333	0.941
Present (n = 115)	1.218			0.989		
*Extrahepatic spread*						
Absent (n = 330)	1	1.018–1.653	0.035	1	0.904–1.510	0.234
Present (n = 192)	1.297			1.169		
*History of sorafenib therapy*						
No (n = 398)	1	1.038–1.741	0.025	1	0.915– 1.571	0.189
Yes (n = 124)	1.344			1.199		
*CAR*						
< 0.108 (n = 302)	1	1.592–2.573	< 0.001	1	1.495–2.452	< 0.001
≥ 0.108 (n = 220)	2.024			1.915		

*HR* Hazard ratio; *CI* Confidence interval; *ECOG-PS* Eastern Cooperative Oncology Group Performance Status; *CAR* C-reactive protein to albumin ratio.

Multivariate Cox proportional hazards modeling with the covariates of age, sex, ECOG-PS, etiology, α-fetoprotein, macroscopic vascular invasion, extrahepatic spread, history of sorafenib therapy, and CAR showed that ECOG-PS ≥ 1 (hazard ratio (HR), 1.429; 95% CI, 1.075–1.899; *p* = 0.014), α-fetoprotein ≥ 400 ng/mL (HR, 1.604; 95% CI, 1.237–2.079; *p* < 0.001), and CAR ≥ 0.108 (HR, 1.915; 95% CI, 1.495–2.452; *p* < 0.001) were independently associated with overall survival (Table 2).

### Factors associated with progression-free survival

Factors associated with progression-free survival in the univariate analysis are listed in Table 3. Etiology and CAR were statistically significantly associated with progression-free survival.

**Table 3:** Progression-free survival analysis.

	Univariate analysis			Multivariate analysis		
	HR	95% CI	*p* value	HR	95% CI	*p* value
*Age (years)*						
< 75 (n = 303)	1	0.841–1.280	0.731	1	0.885–1.372	0.385
≥ 75 (n = 219)	1.038			1.102		
*Sex*						
Female (n = 112)	1	0.851–1.427	0.461	1	0.928–1.593	0.156
Male (n = 410)	1.102			1.216		
*ECOG-PS*						
0 (n = 397)	1	0.978–1.606	0.074	1	0.937–1.570	0.143
≥ 1 (n = 125)	1.253			1.213		
*Etiology*						
Viral hepatitis (n = 291)	1	0.625–0.953	0.016	1	0.583–0.905	0.004
Non-B, non-C (n = 231)	0.772			0.726		
*α-fetoprotein (ng/mL)*						
< 400 (n = 373)	1	0.994–1.555	0.057	1	0.927–1.475	0.186
≥ 400 (n = 149)	1.243			1.170		
*Macroscopic vascular invasion*						
Absent (n = 407)	1	0.765–1.264	0.895	1	0.687–1.172	0.426
Present (n = 115)	0.983			0.897		
*Extrahepatic spread*						
Absent (n = 330)	1	0.921–1.404	0.232	1	0.918–1.423	0.233
Present (n = 192)	1.137			1.143		
*History of sorafenib therapy*						
No (n = 398)	1	0.857–1.369	0.503	1	0.776–1.271	0.956
Yes (n = 124)	1.083			0.993		
*CAR*						
< 0.108 (n = 302)	1	1.291–1.964	< 0.001	1	1.324–2.043	< 0.001
≥ 0.108 (n = 220)	1.592			1.644		

*HR* Hazard ratio; *CI* Confidence interval; *ECOG-PS* Eastern Cooperative Oncology Group performance status; *CAR* C-reactive protein to albumin ratio.

Multivariate Cox proportional hazards modeling with the covariates of age, sex, ECOG-PS, etiology, α-fetoprotein, macroscopic vascular invasion, extrahepatic spread, history of sorafenib therapy, and CAR showed that non-hepatitis B, non-hepatitis C etiology (HR, 0.726; 95% CI, 0.583–0.905; *p* = 0.004) and CAR ≥ 0.108 (HR, 1.644; 95% CI, 1.324–2.043; *p* < 0.001) were independently associated with progression-free survival (Table 3).

### Overall survival and progression-free survival stratified by CAR

**Figure 3:**
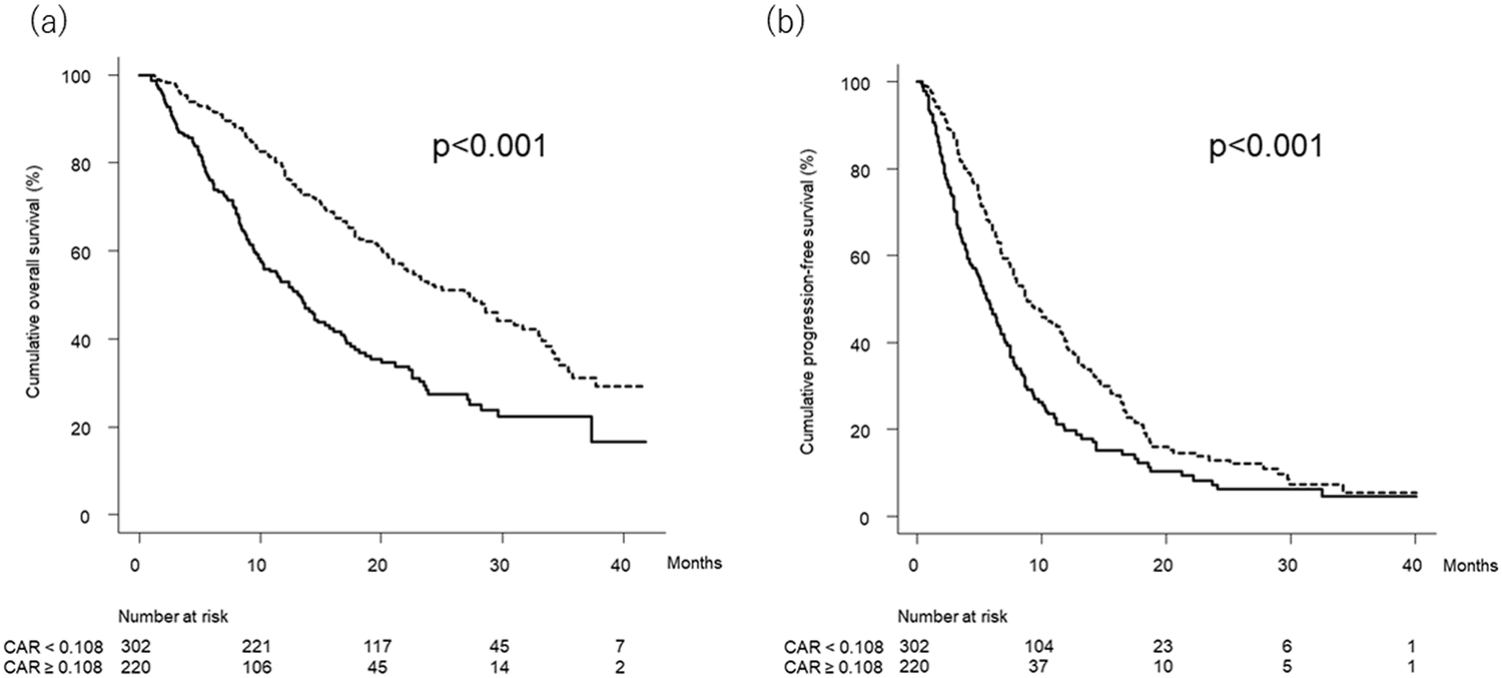
(**a**) Cumulative overall survival curves stratified by CAR. Cumulative overall survival at 6, 12, 18, 24, 30, and 36 months was 92.2%, 78.0%, 63.2%, 53.0%, 44.1%, and 31.1% in Patients with low CAR (< 0.108) (dotted line) and 76.0%, 53.1%, 37.6%, 27.6%, 22.3%, and 22.3% in patients with high CAR (≥ 0.108) (solid line), respectively (*p* < 0.001, log-rank test). (**b**) Cumulative progression-free survival curves stratified by CAR. Cumulative progression-free survival at 3, 6, 9, 12, 15, and 18 months was 87.0%, 67.0%, 49.4%, 39.2%, 29.9%, and 20.9% in patients with low CAR (< 0.108) (dotted line) and 70.5%, 47.7%, 29.2%, 19.7%, 15.2%, and 12.4% in patients with high CAR (≥ 0.108) (solid line), respectively (*p* < 0.001, log-rank test). CAR, C-reactive protein to albumin ratio.

Figure 3a shows the curves for overall survival stratified by CAR. Median overall survival was 27.2 (95% CI, 21.7–31.6) months in patients with low CAR (< 0.108) and 13.3 (95% CI, 10.1–15.7) months in patients with high CAR (≥ 0.108), respectively (*p* < 0.001).

Figure 3b shows the curves for progression-free survival stratified by CAR. Median progression-free survival was 8.8 (95% CI, 7.8–11.2) months in patients with low CAR (< 0.108) and 5.6 (95% CI, 4.5–6.8) months in patients with high CAR (≥ 0.108), respectively (*p* < 0.001).

### Relationship between clinical markers and CAR

**Figure 4:**
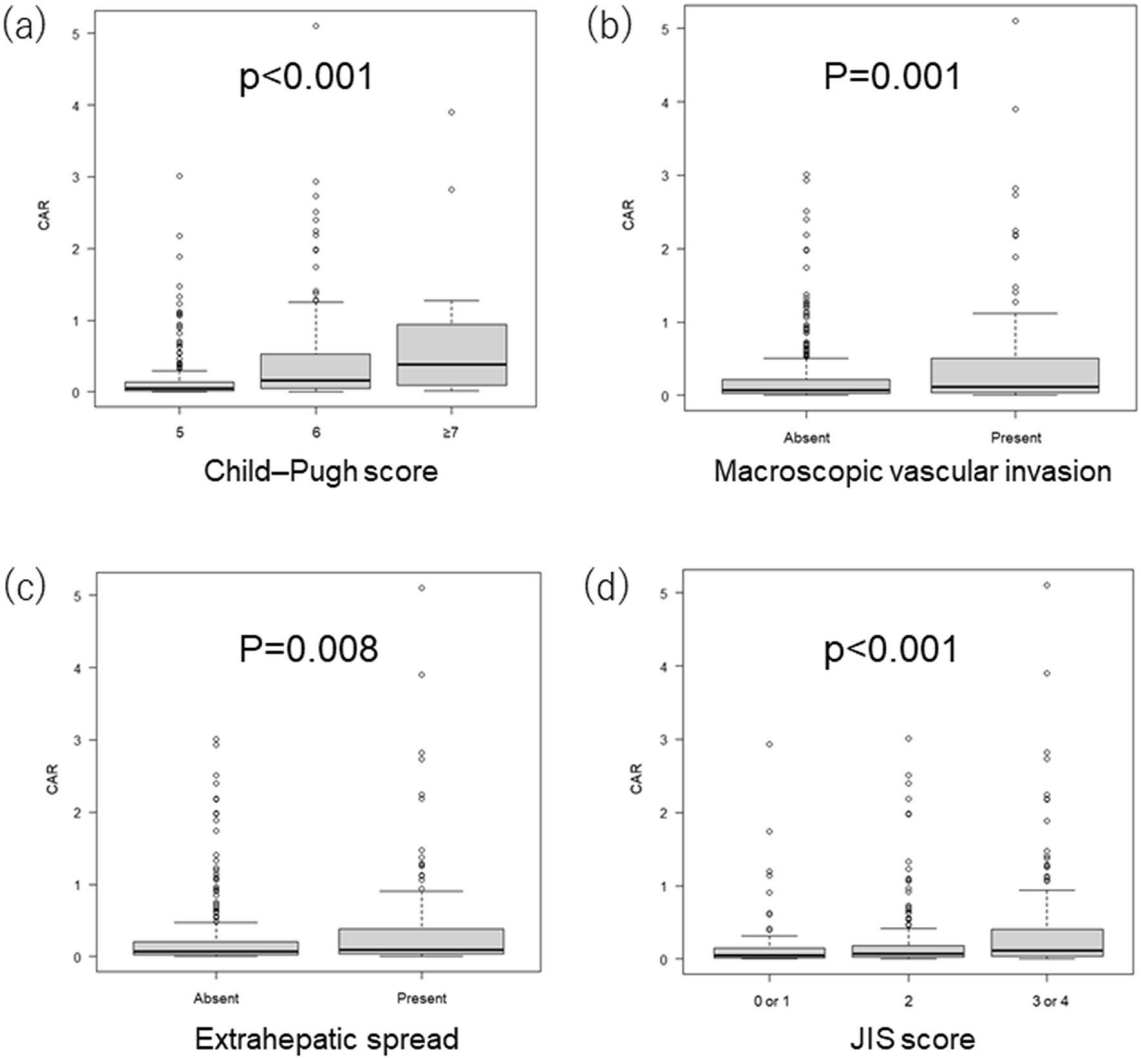
Relationship between clinical markers and CAR. (**a**) Relationship between Child–Pugh score and CAR. CAR values were 0.049 (0.020–0.136), 0.160 (0.047–0.527), and 0.381 (0.133–0.922) in patients with Child–Pugh score of 5 (n = 302), 6 (n = 206), and ≥ 7 (n = 14), respectively (*p* < 0.001, Jonckheere-Terpstra test). (**b**) Relationship between the presence or absence of macroscopic vascular invasion and CAR. CAR values were 0.074 (0.025–0.222) and 0.120 (0.038–0.505) in patients with (n = 407) and without (n = 115) macroscopic vascular invasion, respectively (*p* = 0.001, Mann–Whitney U-test). (**c**) Relationship between the presence or absence of extrahepatic spread and CAR. CAR values were 0.072 (0.025–0.209) and 0.097 (0.036–0.384) in patients with (n = 330) and without (n = 192) extrahepatic spread, respectively (*p* = 0.008, Mann–Whitney U-test). (**d**) Relationship between JIS score and CAR. CAR values were 0.045 (0.015–0.152), 0.074 (0.026–0.188), and 0.116 (0.036–0.409) in patients with JIS score of 0 or 1 (n = 93), 2 (n = 197), and 3 or 4 (n = 232), respectively (*p* < 0.001, Jonckheere-Terpstra test). The box represents the interquartile range. The line through the box indicates the median. The bottom error bar indicates the 25th percentile − 1.5 × interquartile range and the top error bar indicates the 75th percentile + 1.5 × interquartile range. CAR, C-reactive protein to albumin ratio; JIS, Japan Integrated Staging.

Figure 4a shows the relationship between Child–Pugh score and CAR. CAR values were significantly higher as Child–Pugh score increased (*p* < 0.001). Figure 4b shows the relationship between the presence or absence of macroscopic vascular invasion and CAR. CAR values were significantly different between patients with versus without macroscopic vascular invasion (*p* = 0.001). Figure 4c shows the relationship between the presence or absence of extrahepatic spread and CAR. CAR values were significantly different between patients with versus without extrahepatic spread (*p* = 0.008).

Figure 4d shows the relationship between JIS score and CAR. CAR values were significantly higher as JIS score increased (*p* < 0.001).

## Discussion

In this multicenter study, patients with unresectable HCC and treated with lenvatinib who had high CAR had poorer outcomes than patients with low CAR. Multivariate analysis with adjustment for age, sex, ECOG-PS, etiology, α-fetoprotein, macroscopic vascular invasion, extrahepatic spread, history of sorafenib therapy, and CAR showed that CAR is independently associated with overall survival and progression-free survival. In addition, multivariate analysis showed that ECOG-PS and α-fetoprotein are also independently associated with overall survival. Multivariate analysis showed that etiology is also independently associated with progression-free survival. Furthermore, CAR values were significantly higher as JIS score, a useful prognostic factor for HCC, increased. These results suggest that CAR can predict both overall survival and progression-free survival in patients with unresectable HCC treated with lenvatinib. In addition, CAR, a biomarker that is easy and inexpensive to measure, is an integrated marker that reflects hepatic function and HCC stage.

CAR has been investigated as an indicator in digestive malignancies such as esophageal, gastric, colorectal, and pancreatic cancer^11–14^. In HCC, the impact of CAR on survival and recurrence has been reported in patients with HCC treated with surgical resection, radiofrequency ablation, transarterial chemoembolization, or molecularly targeted therapy^15,16^. Oh et al.^15^ investigated the relationship between CAR and outcomes in 389 patients who underwent resection for HCC. They found that a postoperative CAR increase of 1.0 is associated with a 1.171-fold decrease in overall survival (HR, 1.171; 95% CI, 1.072–1.278; *p* < 0.001) and a 1.19-fold decrease in progression-free survival (HR, 1.190; 95% CI, 1.108–1.278; *p* < 0.001) in multivariate analysis^15^. They also found that the optimal CAR cutoff values for predicting overall survival and progression-free survival were 0.625 and 0.500, respectively, using ROC curve analysis^15^. Kinoshita et al.^16^ investigated the relationship between CAR and overall survival in 186 patients with HCC treated with resection, locoregional treatment, transarterial chemoembolization, sorafenib therapy, or best supportive care. They found that CAR ≥ 0.037 (HR, 3.394; 95% CI, 1.986–5.801; *p* < 0.001) was independently associated with worse overall survival in their cohort by multivariate analysis^16^. They determined the optimal CAR cutoff value for predicting overall survival was 0.037 with ROC curve analysis^16^. In this study, we also found that CAR is significantly associated with both overall survival and progression-free survival in multivariate analyses using data obtained from patients with HCC treated lenvatinib, a molecularly targeted agent. An advantage of our study in comparison with the previous studies^15,16^ is that we determined the optimal CAR cutoff value using time-dependent ROC curve analysis for overall survival obtained with the Kaplan–Meier method, not simple ROC curve analysis (i.e., with binary values for survival and death). Another advantage of our study relative to the previous study^16^ is that this study included a large number of patients with HCC treated with lenvatinib, instead of various treatments for HCC.

Accumulating evidence from previous reports indicates that inflammation and malignancy are related. Several mechanisms have been reported for the relationship between inflammatory response and malignancy: (i) tumor growth or invasion could induce tissue inflammation; (ii) necrosis of tumor and hypoxia or local tissue damage might activate an inflammatory response; and (iii) cancer cells, tumor-related leukocytes, or both could induce the production of inflammatory cytokines, such as necrosis factor of tumor, interleukin (IL)-1, IL-6, and IL-8, and vascular endothelial growth factor. These inflammatory cytokines and chemokines facilitate cancer growth, invasion, angiogenesis, metastasis, subversion of the host immune response, and resistance to cytotoxic drugs^17–19^. Among clinical markers of inflammation, CRP is an acute-phase reactant synthesized by hepatocytes that is regulated by proinflammatory cytokines, particularly IL-6^20^. Additionally, the presence of a systemic inflammatory response, evidenced by an elevation of CRP levels, accompanies a decrease in serum albumin concentrations and progressive loss of weight and lean tissue, resulting in poorer ECOG-PS and higher mortality in patients with malignancy^21,22^. This is of particular concern in patients with HCC, given the concomitant underlying illness and possible impaired nutritional status attributed to cirrhosis^23^. In this study, we clarified that high CAR is associated with poor liver function and high tumor burden, such as macroscopic vascular invasion or extrahepatic spread, in patients with HCC treated with a molecularly targeted agent.

The main limitations of present study included its hospital-based subjects and retrospective nature. Although this study included a large number of patients with HCC from multiple liver centers in Japan, further prospective studies with community-based participants and long-term follow-up are warranted. Another limitation of present study was that treatment of HCC after lenvatinib therapy was not analyzed. Because treatment after lenvatinib therapy for HCC might affect prognosis, further studies that include the analysis of HCC treatment after lenvatinib therapy are warranted. Third, there is variation in the literature regarding the cutoff values of CAR for digestive malignancies, including HCC^11–16^. Therefore, it remains problematic to use the CAR cutoff values determined by this study as standardized parameters for patients with unresectable HCC treated with lenvatinib. Further validation studies using the CAR cutoff values determined in this study are warranted. Forth, an intrinsic limitation of CAR is the lack of specificity. In addition, sufficient data for analysis of other inflammatory markers, such as IL-6, were not available from the patients in this study. Finally, in this study, therapeutic response was not evaluated in 41 patients. Because most of the 41 patients for whom a therapeutic response was not available had completed the follow-up period prior to the date of the imaging evaluation.

In conclusion, CAR, a marker that is easy and inexpensive to measure, can predict outcomes in patients with unresectable HCC treated with lenvatinib. In addition, CAR is associated with liver function and HCC progression. Further studies are warranted to confirm these findings in other populations.

## Materials and methods

### Patients

The study protocol was approved by the institutional ethics committee of Ehime Prefectural Central Hospital (IRB No. 30–66) based on the Guidelines for Clinical Research issued by the Ministry of Health, Labour and Welfare of Japan.

**Figure 5:**
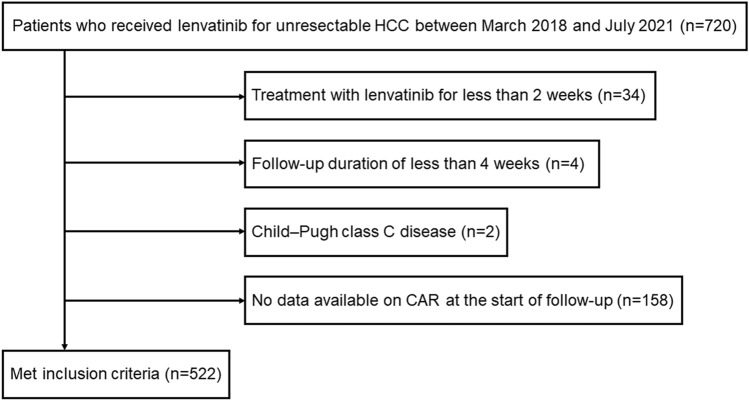
Flowchart of the patient selection process. HCC, hepatocellular carcinoma; CAR, C-reactive protein to albumin ratio.

We enrolled 720 patients with unresectable HCC who received lenvatinib between March 2018 and July 2021 at 20 institutions in Japan. Of these, 522 met the following eligibility criteria: (1) treatment with lenvatinib for more than 2 weeks, (2) follow-up duration of greater than 4 weeks, (3) Child–Pugh class A or B disease, and (4) data available on CAR at the start of follow-up (Fig. 5). CAR was calculated by dividing the CRP level by the albumin level.

We collected clinical data at the start of lenvatinib therapy from the medical records of these 522 patients. The start of follow-up was defined as the date when lenvatinib therapy began. The end of follow-up was defined as the date of the final visit for patients who remained alive or the date of death for patients who died during follow-up.

The etiology of HCC was considered to be hepatitis B virus in patients positive for hepatitis B virus surface antigen. It was considered to be hepatitis C virus in patients positive for hepatitis C virus antibodies.

### Diagnosis and treatment of HCC

HCC was diagnosed based on increases in α-fetoprotein levels, pathological findings, or imaging findings in modalities such as dynamic computed tomography, magnetic resonance imaging, and contrast-enhanced ultrasonography with perflubutane^24,25^. To evaluate tumor progression, we used Barcelona Clinic Liver Cancer (BCLC)^26^ and tumor node metastasis (TNM) staging, determined according to the 6th edition of TNM staging guidelines for HCC by the Liver Cancer Study Group of Japan (LCSGJ) (TNM-LCSGJ)^27^. In addition, we used the JIS system for HCC prognosis^28^. We assessed liver function using the Child–Pugh classification system^29^.

The most appropriate treatment modality for HCC in each patient was selected through discussion between surgeons, hepatologists, and radiologists in each institution, based on Japanese practice guidelines for HCC^30,31^.

### Lenvatinib treatment and adverse events

After obtaining written informed consent from each patient, lenvatinib (Lenvima^®^; Eisai, Tokyo, Japan) treatment was started. The dose of oral lenvatinib was 8 mg/day in patients who weighed < 60 kg and 12 mg/day in patients who weighed ≥ 60 kg. However, in patients with advanced age, non–Child–Pugh A disease, low body weight, and pleural effusion, ascites, or gastrointestinal varices with a risk of bleeding, the initial dose of lenvatinib was reduced at the discretion of the physician.

Lenvatinib was discontinued when any unacceptable or serious adverse event or clinical tumor progression occurred. In accordance with the drug manufacturer’s guidelines, the dose was reduced or treatment was interrupted when a patient developed any grade ≥ 3 severe adverse events or if any unacceptable treatment-related adverse events occurred. Adverse events were assessed using the National Cancer Institute Common Terminology Criteria for Adverse Events, version 5.0^32^. If a treatment-related adverse event occurred, dose reduction or temporary interruption occurred until the symptom resolved to grade 1 or 2, based on the manufacturer’s guidelines.

### Evaluation of therapeutic response

Local physicians at each institution evaluated tumors using enhanced computed tomography or magnetic resonance imaging at 4 or 12 weeks after introducing lenvatinib, in accordance with the modified Response Evaluation Criteria in Solid Tumors^33,34^.

### Statistical analysis

Continuous variables are expressed as medians (interquartile range). The Mann–Whitney U-test was used for continuous variables. The χ^2^-test or Fisher’s exact test was used for categorical variables. In addition, the Jonckheere-Terpstra test was used to analyze trends between CAR values and clinical markers.

Actuarial analysis of cumulative overall survival and progression-free survival was performed using the Kaplan–Meier method, and differences were assessed with the log-rank test. Univariate and multivariate Cox proportional hazards models were used for analysis of factors related to overall survival and progression-free survival.

Time-dependent ROC curves for overall survival were obtained with the Kaplan–Meier method by CAR^35^. We determined the cutoff value for CAR at the median overall survival of this study cohort using the maximum Youden index (sensitivity + specifcity − 1)^36^. In this study, we used age of 75 years and α-fetoprotein of 400 ng/mL as cutoff values for analysis based on previous report^37^.

Statistical significance was defined as *p* < 0.05. Statistical analyses were performed with EZR Ver. 1.53 (Saitama Medical Center, Jichi Medical University, Saitama, Japan), which is a graphical user interface for R (The R Foundation for Statistical Computing, Vienna, Austria)^38^. More precisely, it is a modified version of the R commander designed to add statistical functions frequently used in biostatistics.

### Ethics approval

The protocol used in the present study was approved by the Institutional Ethics Committee of Ehime Prefectural Central Hospital (IRB No. 30-66), based on the Guidelines for Clinical Research issued by the Ministry of Health and Welfare of Japan. All methods were carried out in accordance with relevant guidelines and regulations.

### Consent to participate

Written informed consent was obtained from each patient.

### Consent for publication

Written informed consent was obtained from each patient.

## Data Availability

The datasets are available from the corresponding author on reasonable request.

## Abbreviations

HCC: Hepatocellular carcinoma
ECOG-PS: Eastern Cooperative Oncology Group performance status
CRP: C-reactive protein
CAR: C-reactive protein to albumin ratio
BCLC: Barcelona Clinic Liver Cancer
TNM: Tumor node metastasis
LCSGJ: Liver Cancer Study Group of Japan
JIS: Japan Integrated Staging
ROC: Receiver operating characteristic
CI: Confidence interval
HR: Hazard ratio
IL: Interleukin
